# Test-Retest Reliability of Ultrasonographic Measurements from the Rectus Femoris Muscle 1–5 Years after Anterior Cruciate Ligament Reconstruction in the Ipsilateral and Contralateral Legs: An Observational, Case-Control Study

**DOI:** 10.3390/jcm11071867

**Published:** 2022-03-28

**Authors:** Jorge Buelga-Suarez, Pablo Alba-Martin, Nicolas Cuenca-Zaldívar, María García-Escudero, Pilar Bierge-Sanclemente, Jaime Almazán-Polo, Samuel Fernández-Carnero, Daniel Pecos-Martín

**Affiliations:** 1 Clinica Premium, 28016 Madrid, Spain; jorgebuelga@hotmail.com (J.B.-S.); whitepaopu@hotmail.com (P.A.-M.); 2 Functional Recovery Unit, Hospital Guadarrama, 28440 Guadarrama, Spain; nicolas.cuenca@salud.madrid.org; 3 Research Group in Nursing and Health Care, Puerta de Hierro Health Research Institute—Segovia de Arana (IDIPHISA), 28222 Madrid, Spain; 4 School of Health Sciences, Universidad Católica de Valencia San Vicente Mártir, 46900 Valencia, Spain; maria.escudero@ucv.es; 5 Clínica Spai de Salut, 46200 Valencia, Spain; pilarbierge@gmail.com; 6 Faculty of Sport Sciences, Universidad Europea de Madrid, 28670 Madrid, Spain; jaime.almazan@universidadeuropea.es; 7 Universidad de Alcalá, Facultad de Enfermería y Fisioterapia, Departamento de Fisioterapia, Grupo de Investigacion en Fisioterapia y Dolor, 28801 Alcalá de Henares, Spain; daniel.pecos@uah.es

**Keywords:** knee injuries, arthroscopy, ultrasonography, quadriceps muscle, muscle contraction

## Abstract

About 40% of traumatic injuries in sports are related to the knee. Of these, 33% require arthroscopic surgery. The rehabilitative ultrasound imaging technique is a simple method to obtain objective real-time results on the state and measurement of the musculoskeletal tissue and its use can represent an important change in the process of functional diagnosis and recovery of these injuries. The aim was to quantify the differences in the thickness, muscle contraction time, and muscle relaxation time of the rectus femoris muscle between individuals with knee arthroscopy and healthy individuals and to verify the reliability of the inter-examiner measurements in these ultrasound variables. An observational case-control study with individuals (18–60 years aged) who underwent surgery for anterior cruciate ligament through knee arthroscopy a year or more before. A total of 38 subjects were divided into 2 groups, case and control. Ultrasound measurements were taken of the following outcomes: thickness at rest and contraction, muscle contraction time, and muscle relaxation time of the rectus femoris muscle. Excellent inter-examiner reliability was obtained for all ultrasound measurements (ICC3.3 > 0.90). No significant changes were found in the rate of contraction or rest of the rectus femoris muscle. On the other hand, if significant changes in the thickness of the rectus femoris muscle were found between control and case group. Arthroscopic surgery for anterior cruciate ligament reconstruction does not appear to modify function but does modify the thickness of the rectus femoris muscle on ultrasound examination. Ultrasound appears to be a reliable tool for the study of these measurements in the rectus femoris muscle.

## 1. Introduction

At present, the amount of time spent doing sports has increased significantly due to a growing awareness of the beneficial effects of physical activity on health and a greater awareness of healthy lifestyles [1]. The number of people practicing sports has increased in the last decade, as well as the number of people federated in different sports disciplines [2]. Lower limb injuries are the most pronounced in sports that have experienced a boom in recent years, especially in high-speed sports and team sports such as snowboarding, mountain biking, or skating. These lower limb injuries, more specifically knee injuries, are the main reason for sports sick-leave in soccer, basketball, and sports commonly practiced in European society [3,4].

Despite the importance of the knee joint in the functional performance of the lower limb and, therefore, in sports performance, scant literature is found on the prevalence of knee injuries in sports and their complications [5].

About 39% of traumatic injuries in sports are of the knee. These injuries are divided into muscle injuries, tendon injuries, contusions, internal knee trauma (meniscopathies), cartilage injuries, and ligament injuries. A total of 44% of knee pathologies are compatible with internal knee trauma, and 33% end up with arthroscopic surgery [5].

Musculoskeletal evaluation in the world of health sciences has always been a constant as a predictive value of injury and a related factor to be considered after an injury. Several studies certify the assessment by ultrasonography as a method to obtain information on the cross-sectional muscle section [6,7,8,9].

However, several more recent studies seem to indicate that there are other parameters that can be measured with musculoskeletal ultrasound that are also reliable for determining the functional capacity of the muscle, such as the contraction and resting velocity of the muscle [10,11,12].

Musculoskeletal ultrasound scanning is a simple method of obtaining objective real-time results on the condition and measurement of skeletal muscle tissue. The standard test used is and has so far been magnetic resonance imaging; however, more and more research is being carried out with ultrasonography [13,14,15]. The most studied regions have been the abdominal and lumbar multifidus musculature, although the RUSI (rehabilitative ultrasound imaging) technique has been used in lower limb musculature such as the vastus internus of the quadriceps muscle in comparison with the MRI (nuclear magnetic resonance) [6,14,15].

The RUSI technique used by physiotherapists recognizes them as the health professionals to carry out this type of intervention [7].

In view of the above and considering the high prevalence of knee injury, the health care costs involved in performing arthroscopy, and the complications that this intervention may cause, it is considered important to explore the possible changes in muscle function associated with this intervention. By means of ultrasonography, we can obtain objective data on the state of the musculoskeletal tissue in real time, which can facilitate the evolution of the patients. Therefore, the aim of the study was to quantify the differences in the thickness and speed of contraction and relaxation of the rectus femoris muscle (RFM) between healthy(control) and anterior cruciate ligament (ACL)-operated individuals (case), as well as to check the inter-examiner reliability of the ultrasound variables measurements in order to answer the hypothesis that behavior and morphology of rectus femoris observed by ultrasound, detailed in the aim in this patients, could remain altered in 1–5 years follow-up.

## 2. Materials and Methods

### 2.1. Design

A cross-sectional observational case-control study was designed. The recommendations of the STROBE initiative for observational studies were followed, as well as the rules described in the Helsinki Declaration in its revision text as of 5 April 2021, and the rules for the treatment of personal data according to Organic Law 15/1999 on the protection of personal data, and European Regulation 2016/679 [16,17].

This study was approved by the Ethics Committee of the University of Alcalá (CEIM2021/04/097).

### 2.2. Participants

A total of 38 subjects participated in the study after voluntarily signing the informed consent form. Recruitment was performed at the “Premium Madrid Rehabilitation Center” (Madrid, Spain) from patients who underwent an ACL rehabilitation program. The sample was divided into two groups: the case group, formed by subjects of both sexes who had undergone surgery for ACL ligamentoplasty by knee arthroscopy with the 4-stranded hamstring tendons graft method and with no other previous history of musculoskeletal diseases in the last year, and the control group, formed by healthy individuals with demographic, anthropometric, and physical activity variables such as those of the case group, thus ensuring homogeneity between the two groups.

The following inclusion criteria were established: for the case group, it was an indispensable condition to have been at least one year after surgery. For both groups, the inclusion criteria were established as being between 18 and 60 years of age, the absence of pain in the lower limb, not presenting any limitation in the articular range of the knee, and habitually performing physical activity involving impact exercise with a score of at least “moderate” on the International Physical Activity Questionnaire (IPAQ) [18]. In addition, all subjects who participated in the study were required to read and freely sign the informed consent form.

As exclusion criteria for the case group, it was determined to exclude those subjects who had undergone bone-tendon-bone surgery or who had suffered any post-surgical complication such as infections, coagulation problems, ACL re-rupture, or neurological lesions. For both groups, refer to previous medical-surgical history in the lower extremities, such as fractures, ligament ruptures, arthroplasties, and/or be diagnosed with any systemic disease that may produce musculoskeletal alterations or changes in the subcutaneous and muscular cellular tissue, such as diabetes, rheumatoid arthritis or hypothyroidism [19,20]. Subjects with excessive muscle volume that made reliable ultrasound measurement impossible were also excluded [14].

### 2.3. Sample Size

A sample size of 38 subjects was estimated, 19 in the case group and 19 in the control group, to detect a difference equal to or greater than 3.6 mm [20] in the thickness of the RFM as the minimum detectable change and assuming a common standard deviation of 4.8 mm [21], accepting an alpha risk of 0.05 and beta risk of 0.2 in unilateral contrast. The Granmo sample size calculator, version 7.12 (Instituto Hospital del Mar de Investigaciones Médicas, Barcelona, Spain), was used.

#### 2.3.1. Measurements

High-quality ultrasonography equipment (Ecube i7; Alpinion Medical System; Seoul, Korea) was used to perform all ultrasound imaging with a linear probe L3_12T with a frequency range of 8 to 12.0 MHz, and a 45 mm footprint was used to perform the resting and contraction measurements of all variables to be studied. B-mode ultrasound imaging, with settings determined to obtain an image, allowed measurements to be made both at rest and in contraction.

**Figure 1:**
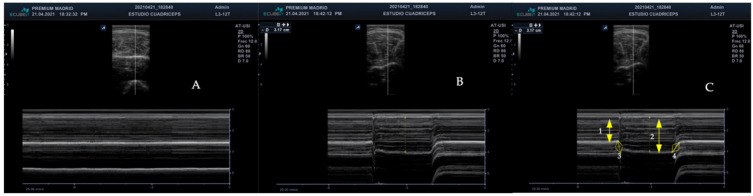
M-mode RFM measurements. Muscle at rest (**A**), muscle at contraction (**B**), and muscle at contraction with measurements (**C**) where: rest (1), contraction (2), muscle contraction time (3), and muscle relaxation time (4).

**Figure 2:**
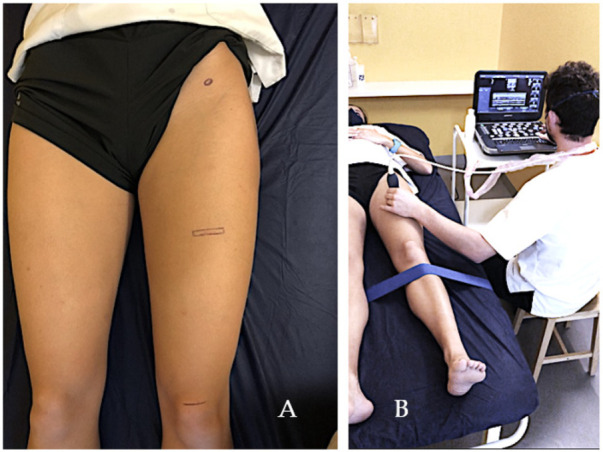
Researcher and patient preparations and positioning. Surface references (**A**) and ultrasound assessment sampling (**B**).

The main outcome variables were measured in the ultrasound machine as follow: (1) muscle contraction time of the RFM of both legs (cm/s) and muscle relaxation time of the RFM of both legs (cm/s) (Figure 1A); (2) thickness of the RFM of both legs at rest and contraction (mm) (Figure 1B,C).

Other descriptive, demographic and anthropometric variables were collected: age, sex (male = 0; female = 1), weight (kg), height (cm), body mass index (BMI), (kg)/height^2^ (m); categorical qualitative variables: time elapsed from injury to surgery (1–5 years = 0; +5 years = 1); type of injury (ACL = 0; ACL + internal meniscus (IM) = 1; ACL + external meniscus (EM) = 2; ACL + IM + medial collateral ligament (MCL) = 3); type of intervention performed (autograft = 0; halograft = 1); treatments performed after the intervention (nothing = 0; physiotherapy = 1; physiotherapy + exercises = 2), IPAQ (high = 1; moderate = 2; low = 3).

In relation to the naming and management of personal data, a history number was randomly assigned to each participant. This was the identifying element of each participant, along with anthropometric and clinical data to ensure the privacy of the information, to respect the General Data Protection Regulation (GDPR, EU) 2016/679 of the European Parliament and of the Council of 27 April 2016 on the protection of natural persons with regard to the processing of personal data and on the free movement of such data and Law 14/2007, on biomedical research on human beings.

To mask as much as possible the results of the study, the researchers responsible for sample selection, subject assessment, and data collection and analysis will be independent of each other.

A first researcher was designated to perform the anamnesis and physical examination to verify that the subject met the requirements to form part of the groups that made up the study (first data collection). Likewise, this researcher informed, in writing and verbally, of all those aspects of the study that were of interest to the subjects and requested duly completed and signed informed consent to participate in the study. Each study subject was randomly assigned a medical history number to mask personal data.

This researcher, with the data collected in the first data collection, distributed the participants in each group and oversaw arranging the appointments for the measurements.

Two researchers were designated who consecutively carried out the ultrasound measurements of all the main variables of the study on the subjects. These measurements were performed in a single session. Ultrasound measurements were taken according to the following procedure: Subject in supine position with hip in neutral position and knee extended, a rigid tape is placed over the tibia to block knee flexion. The probe is placed over the middle third of the quadriceps, finding in the image the RFM. A transverse section was made over the previously marked area. (Figure 2A). The subject was asked to push with the back of the knee against the stretcher and slightly elevate the lower limb, a maximum isometric contraction in knee extension and hip flexion of 3 s duration, and 3 images were taken of 3 different contractions at rest and in contraction, using mode M. The procedure was repeated on both legs.

Verbal cues to the patient were to hold the maximum contraction for 3 s, extend the knee to touch the back of the knee to the stretcher, and lift the leg straight.

Each study participant was assigned a code during all measurements, and the two researchers performing the ultrasound measurements were blinded to each other, performing the measurements in separate booths.

A fourth researcher, external to the data collection, sample selection, and measurement of the variables, will perform the statistical analysis of the data collected.

#### 2.3.2. Statistical Analysis

The normality of the continuous variables was evaluated with the Shapiro–Wilk test, fulfilling all the assumptions. For the descriptive analysis of quantitative variables, the mean and standard deviation (SD) were used, and for categorical variables, absolute frequencies and percentages were used. The analysis of the homogeneity of the groups in the quantitative variables was carried out using the Student’s *t*-test and for the categorical variables using Pearson’s chi-square test [22,23,24].

For the analysis of inter-examiner reliability, the intraclass correlation coefficient under mixed model and absolute agreement for the mean of 3 measurements (ICC3.3) was used. We also calculated the standard error of the mean (SEM) as the square root of the mean square of the analysis of variance (ANOVA) error and the minimum detectable difference at 95% confidence (MDC95) with the formula SEM × 2 – √ × 1.96SEM × 2 × 1.96. SEM and MDC95 were also reported as a percentage with respect to the sample mean. Assessment of compliance with the homoscedasticity assumption was assessed by visual inspection of the Bland–Altman plots [25].

For the analysis of the differences between the groups in the thickness of the RFM, 2-by-2 mixed analyses of covariance (ANCOVA) were used separately for each side (operated and non-operated), with the muscle state factor (rest, contraction) as an intra-subject factor and the group factor (case/control) as an inter-subject factor. For the control group, the mean of both sides was used. The age and BMI of the subjects were included as covariates. The size of the main effects and interactions of the ANCOVA was assessed with the partial eta squared coefficient (ηp2), with 0.01 being a small size, 0.06 a medium size, and 0.14 a large size [23,24,25]. Post-hoc pairwise comparisons were performed with the Student’s *t*-test with Bonferroni correction [22].

Finally, for the analysis of between-group differences in contraction and relaxation velocity (for the control group, the mean of both sides was used) for the operated and non-operated sides, multiple linear regression analyses were used, introducing the group variable as predictor and age and BMI as covariates, to find the between-group differences adjusted for the covariates [22,26,27].

All analyses were performed with the R 4.1.0 software (R Core Team (2021). R: A language and environment for statistical computing. R Foundation for Statistical Computing, Vienna, Austria. URL https://www.R-project.org/, accessed on 1 October 2021).

## 3. Results

**Figure 3:**
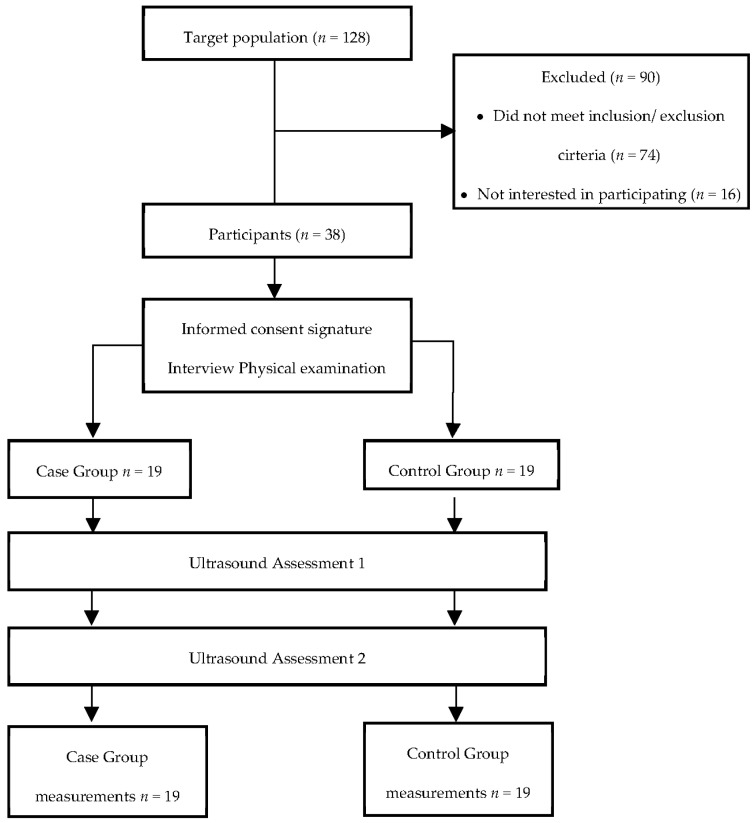
Flow chart diagram.

The final sample consisted of 38 subjects (Figure 3). The demographic characteristics are shown in Table 1.

### 3.1. Inter-Examiner Reliability

Excellent inter-examiner reliability was obtained for all ultrasound measurements (ICC3,3 > 0.90) (Table 2).

### 3.2. Differences in Muscle Thickness

The 2-by-2 mixed ANCOVA for muscle thickness on the operated side found a significant main effect for the muscle condition factor (F = 7.33; *p* = 0.01; ηp2 = 0.18) and for the group factor (F = 7.28; *p* = 0.01; ηp2 = 0.18). No significant state-by-group interaction was found (F = 0.06; *p* = 0.81; ηp2 = 0.002). However, for the non-operated side, the mixed ANCOVA showed a significant main effect for the muscle condition factor (F = 17.52; *p* < 0.01; ηp2 = 0.33), but not for the group factor (F = 0.29; *p* = 0.59; ηp2 = 0.01). Nor was a significant state-by-group interaction found (F = 41; *p* = 0.53; ηp2 = 0.01). Pairwise comparisons are reflected in Table 3.

### 3.3. Differences in Contraction and Relaxation Speed

Multiple linear regression analyses showed no differences adjusted for age and BMI between control and operated subjects, in contraction and relaxation speed, for either side, operated and non-operated (Table 4).

## 4. Discussion

This study aimed to measure and compare the contraction velocity, return-to-calm (relaxation) speed, and thickness of the RFM, assuming that it was lower in individuals who had undergone ACL ligamentoplasty for more than one year compared to control individuals who had not undergone surgery. The findings of this research do not reflect significant differences in some of these values between both groups of individuals. Significant differences were observed between both lower limbs in the operated subjects.

These findings suggest that at least one year after this type of surgery, the quadriceps muscle is able to reach its maximum contraction and return to rest with the same speed as in individuals who have not undergone this type of arthroscopic intervention. This indicates that one of the basic functional capacities of skeletal muscle is perfectly recoverable despite the traumatic event of arthroscopic knee surgery. However, the thickness of the musculature, which is indicative of muscle volume, is significantly affected, being less on both contraction and at rest on the operated side than on the non-operated side [28]. Furthermore, on the non-intervened side, there were no differences with control subjects.

Ultrasound scanning has demonstrated in patients with low back pain after the episode resolution changes in paravertebral muscles thickness [29], giving clinicians the opportunity to take another clinical approach even in 10 weeks follow-up, and our study has found coincidence with this previous evidence; thus, 1 year after surgery, the differences still remain in ACL surgery patients.

Normal reference for lumbar region has been also detailed in literature for better understanding in ultrasound scanning [30,31], giving exploratory keys in actual clinical settings with patients with chronic lumbar pain. The results of muscle thickness reported in RFM ultrasound in this study has looked for the normal values in healthy people (2.26 ± 0.05) and those after ACL surgery (2.08 ± 0.05) with a (CI −0.17 (−0.33 to −0.02)), setting a precedent for assessment that could be helpful in the clinical setting.

Lower limbs muscles have also been studied, and the relation between muscles thickness and pre-existence of pathology has been studied previously in the chronic ankle sprains [32], the correlation of muscle thickness in the fibular musculature compared to healthy individuals (controls 0.5 ± 0.2 (0.2–1.2) cases 0.4 ± 0.2 (0.1–1.1) *p*-value 0.002) becomes in a very similar to that achieved in our study of the rectus femoris quadriceps in ACL surgeries.

Even diaphragm muscles have been correlated in contraction and rest in patients with low back pain [33], observing in controls (0.16 ± 0.07) cases (0.23 ± 0.06) *p*-value 0.006) also coincident with the results, or our study.

The use of ultrasound in physiotherapy as a musculoskeletal assessment has increased [34,35] last decades, and in this study, we verified in an analytical and isolated way the decrease in thickness both in contraction and at rest (Table 3) of the anterior rectus of the quadriceps of the subjects operated on for ACL.

Finally, regarding validity and reliability, the intraclass correlation was analyzed by the inter-examiner, obtaining excellent results (ICC 0.99 (CI 0.99–1.00)) for resting thickness and contraction too (Table 2) and for M-mode too (ICC 0.99 (0.98–1.00)) as published in previous studies.

The present study could have several limitations such as sample analyzed but demonstrated a low level of heterogeneity regarding the sex and weight variables (*p* < 0.005) could be considered a limitation, but do not consider could affect the results. The need for a sample with such specific criteria may limit the selection of cases since it may be difficult to find individuals with these characteristics who are at the desired time of post-surgical evolution to carry out the study. Likewise, statistically significant differences could be found between individuals who have exceeded 5 years of evolution after surgery and those who have not, so this has been chosen as a qualitative numerical variable to be considered.

The training modality and the different types of sports activities to which the subjects are exposed on a daily basis could have a significant effect on the results obtained in the measurement of the main variables. This fact may limit comparisons between study subjects [36].

Despite the fact that the use of musculoskeletal ultrasound as a reliable instrument to measure the contraction and resting speed of the RFM has been demonstrated in previous studies, to the best of our knowledge, we have not been able to find studies that have proven inter-examiner reliability, which is why it was decided to establish as an objective within the present investigation [10,12,14,37,38,39].

Other studies are necessary to assess the rest of the quadriceps muscles and verify possible adaptations of the vastus medialis, vastus lateralis, or vastus intermedius post-surgery.

Considering all this previous literature and seeing the similarities with our results, it looks the muscle activity and the resting ultrasound scanning could be co-related to the previous pathology or existence and could be recommended the ultrasound evaluation for total patients’ recovery or enhancing healthcare in patients with ACL surgery.

Future research that performs this type of ultrasound measurements prior to surgery and one year after surgery could provide more data regarding the degree of involvement of the muscles after arthroscopic surgery. Likewise, it could be interesting to perform other types of measurements, such as the strength of the RFM, to try to determine if a decrease in muscle thickness leads to a decrease in the strength of this muscle or if the recovery of the original contraction and rest speed is sufficient to maintain the optimal functional state of the skeletal muscle [38]. Based on the observations and results of these investigations, it will be possible to determine with greater evidence what the best approach to rehabilitation treatment after this type of surgery may be.

## 5. Conclusions

The results of this study indicate that there are no significant differences in the contraction or relaxation speed between control individuals and individuals who have undergone arthroscopic knee surgery for ACL reconstruction at least one year after the surgery. On the other hand, significant differences have been obtained in the muscle thickness of the RFM in the operated individuals with respect to their non-operated limb, both in the resting state and in the muscle contraction state. In the control lower limb, no significant differences were found between the control group and the case group.

Regarding the inter-examiner reliability of the ultrasound measurements, the results indicate that ultrasound, used as a method of morphofunctional study at rest and in contraction, is a reliable tool for the exploration of the RFM. Nevertheless, further case studies in this line of research are considered necessary to help generalize the results obtained in the present study.

## Figures and Tables

**Table 1:** Demographics characteristics.

Variable	Control (*n* = 19)	Case (*n* = 19)	*p*-Value
Age, years	33.84 (7.00)	32.47 (9.01)	0.60
Height, cm	170.21 (7.83)	173.47 (7.96)	0.21
Weight, kg	69.21 (7.55)	74.89 (9.09)	0.04
BMI, kg/m^2^	20.31 (1.78)	21.56 (2.18)	0.06
Sex, woman (%)	9 (47.4)	3 (15.8)	0.04
Dominant side, *n* (%)			
Dominant	16 (84.2)	3 (15.8)	
Non-dominant	16 (84.2)	3 (15.8)	
Operated side, *n* (%)			
Right	11 (57.9)	-	
Left	8 (42.1)	-	
Associated injury, *n* (%)			
ACL	15 (39.5)	-	
ACL + IM	2 (10.5)	-	
ACL + EM	2 (10.5)	-	
Time, years, median (IQR)	2 (1.00–3.50)		
IPAQ			0.10
High	8 (41.1)	13 (68.4)	
Moderate	11 (57.9)	6 (31.6)	
Low	0	0	

Data are presented as mean (standard deviation) unless otherwise specified. Abbreviations: cm (centimeters), kg (kilograms), BMI (body mass index), ACL (anterior cruciate ligament), IM (internal meniscus), EM (external meniscus), IPAQ (International Physical Education Questionnaire).

**Table 2:** Reliability of ultrasound measurements.

Variable	Examiner 1 *	Examiner 2 *	ICC_3,3_ (95% CI)	SEM (%)	MDC95 (%)
**Resting thickness**					
Right	2.24 (0.24)	2.23 (0.24)	0.99 (0.99–1.00)	0.03 (1.16%)	0.07 (3.21%)
Left	2.26 (0.24)	2.24 (0.24)	0.99 (0.98–1.00)	0.03 (1.21%)	0.08 (3.37%)
**Contraction thickness**					
Right	2.72 (0.17)	2.71 (0.17)	0.99 (0.99–1.00)	0.01 (0.46%)	0.03 (1.28%)
Left	2.69 (0.17)	2.68 (0.17)	0.99 (0.99–1.00)	0.01 (0.33%)	0.02 (0.90%)
**Muscle relaxation time**					
Right	0.98 (0.32)	0.99 (0.34)	0.99 (0.96–1.00)	0.06 (5.78%)	0.16 (16.01%)
Left	0.89 (0.38)	0.91 (0.37)	0.99 (0.98–1.00)	0.05 (5.54%)	0.14 (15.36%)
**Muscle contraction time+**					
Right	1.12 (0.37)	1.13 (0.38)	0.99 (0.97–1.00)	0.06 (5.38%)	0.17 (14.92%)
Left	1.02 (0.45)	1.05 (0.43)	0.99 (0.98–1.00)	0.05 (5.22%)	0.15 (14.47%)

* Mean (standard deviation). Abbreviations: ICC, intraclass correlation coefficient; CI, confidence interval; SEM, standard error of the mean; MDC95, minimum detectable difference at 95% confidence.

**Table 3:** Differences adjusted for muscle thickness.

State	Control ^#^	Case ^#^	Difference, Mean (CI 95%)
**Operated side**			
Rest	2.26 ± 0.05	2.08 ± 0.05	−0.17 * (−0.33 to −0.02)
Contraction	2.69 ± 0.05	2.50 ± 0.05	−0.19 * (−0.32 to −0.05)
Difference, mean (CI 95%)	0.43 * (0.36 to 0.50)	0.42 * (0.35 to 0.49)	
**Non-operated side**			
Rest	2.26 ± 0.05	2.22 (0.04)	−0.04 (−0.17 to 0.09)
Contraction	2.69 ± 0.05	2.63 (0.04)	−0.06 (−0.19 to 0.07)
Difference, mean (CI 95%)	0.44 * (0.37 to 0.51)	0.41 * (0.34 to 0.48)	

^#^ Data are presented as mean ± standard error of the mean.* Statistically significant (*p* < 0.05). Abbreviations: CI, confidence interval.

**Table 4:** Adjusted differences in contraction and relaxation velocity.

State	Control ^#^	Case ^#^	Difference, Mean (CI 95%)
**Operated side**			
Contraction	1.06 ± 0.08	0.92 ± 0.08	−0.15 (−0.38 to 0.09)
Rest	0.93 ± 0.07	0.88 ± 0.08	−0.04 (−0.26 to 0.17)
**Non-operated side**			
Contraction	1.07 ± 0.08	1.02 ± 0.08	−0.07 (−0.31 to 0.17)
Rest	0.93 ± 0.07	0.89 ± 0.07	−0.04 (−0.25 to 0.17)

^#^ Data are presented as mean ± standard error of the mean. Abbreviations: CI, confidence interval.

## Data Availability

The data sets used and/or analyzed in the current study or any query regarding the research process are available from the corresponding author.
